# Zoonotic Origins of Human Metapneumovirus: A Journey from Birds to Humans

**DOI:** 10.3390/v14040677

**Published:** 2022-03-25

**Authors:** Sonja T. Jesse, Martin Ludlow, Albert D. M. E. Osterhaus

**Affiliations:** Research Center for Emerging Infections and Zoonoses, University of Veterinary Medicine Hannover, 30559 Hannover, Germany; sonja.tatjana.jesse@tiho-hannover.de (S.T.J.); martin.ludlow@tiho-hannover.de (M.L.)

**Keywords:** avian metapneumovirus, human metapneumovirus, host tropism, virus evolution, animal models, F protein, G protein

## Abstract

Metapneumoviruses, members of the family Pneumoviridae, have been identified in birds (avian metapneumoviruses; AMPV’s) and humans (human metapneumoviruses; HMPV’s). AMPV and HMPV are closely related viruses with a similar genomic organization and cause respiratory tract illnesses in birds and humans, respectively. AMPV can be classified into four subgroups, A–D, and is the etiological agent of turkey rhinotracheitis and swollen head syndrome in chickens. Epidemiological studies have indicated that AMPV also circulates in wild bird species which may act as reservoir hosts for novel subtypes. HMPV was first discovered in 2001, but retrospective studies have shown that HMPV has been circulating in humans for at least 50 years. AMPV subgroup C is more closely related to HMPV than to any other AMPV subgroup, suggesting that HMPV has evolved from AMPV-C following zoonotic transfer. In this review, we present a historical perspective on the discovery of metapneumoviruses and discuss the host tropism, pathogenicity, and molecular characteristics of the different AMPV and HMPV subgroups to provide increased focus on the necessity to better understand the evolutionary pathways through which HMPV emerged as a seasonal endemic human respiratory virus.

## 1. Zoonotic Virus Infections: Learning from the Past

The ongoing global health crisis caused by the SARS-CoV-2 pandemic has unequivocally reminded us of the impact that a newly emerging zoonotic respiratory virus can have on a naïve human population. Although the origin of SARS-CoV-2 remains to be established [1], an evolutionary ancestor is most likely present in a rhinolophid bat reservoir [2,3]. A critical question from a future perspective is how long and with what societal impact will SARS-CoV-2 continue to manifest itself in the human population [4,5]. Influenza viruses responsible for previous pandemics in the past century have continued to circulate as seasonal respiratory viruses for decades [6]. In this light, it is interesting to consider the postulated pandemic and bovine origin of Human coronavirus OC43, a SARS-CoV-2 related beta-coronavirus that currently causes moderate seasonal respiratory infections, predominantly among high-risk groups [7]. This has led to speculation that SARS-CoV-2 may also become a seasonal respiratory virus with lower levels of morbidity and mortality, although when that will happen is impossible to predict [8]. Similarly, the origin of most other endemic human respiratory viruses with a major impact on human health and society can eventually be traced back to past zoonotic events [9]. The identification of closely related animal viruses has already illuminated the evolutionary origins of several human viruses which have a respiratory route of transmission including influenza, measles, mumps, and rubella viruses [10,11,12,13].

Human metapneumovirus (HMPV) is also assumed to have evolved following a zoonotic virus infection from an unknown avian reservoir host species, as this virus shares a common ancestor with avian metapneumovirus subtype C (AMPV-C) [14]. Analysis of metapneumovirus sequences using Bayesian phylogeny has indicated that a spill-over event from an avian reservoir may have occurred approximately 200 years ago [15]. However, this leaves many open questions including the possibility of an intermediate host species, which molecular determinants influenced virus adaptation to the human host, and why, with the ever-expanding use of metagenomics, no metapneumoviruses have been identified in any other mammalian species. Our understanding of the global disease burden caused by HMPV infections has improved in recent years with the annual rate of hospitalization associated with HMPV in children younger than five years of age reported being 1 per 1000 [16]. In addition, the PERCH study, which documented the most common cause of severe pneumonia in hospitalized children in seven countries in Africa and Asia over the course of two years, has reported viruses (61%) to be the most common cause [17]. HMPV is ranked third (7.5%) behind respiratory syncytial virus (RSV) (31.1%) and Rhinoviruses (7.5%) as a cause of severe childhood pneumonia in cumulative statistics at all sites, age groups, and severities [17]. In the present review, we discuss the current knowledge about the discovery, evolution, key molecular determinants, and host range of both avian and human metapneumoviruses. Collectively, this analysis provides context to previous and postulated cross-species infections of animal metapneumoviruses.

## 2. Discovery of Avian Metapneumovirus

Intensive research efforts were focused in the 1970s on determining the etiological origins of a well-known disease complex in commercial turkey poults (*Meleagris gallopavo*) termed “acute respiratory disease syndrome”, or most commonly known as “turkey rhinotracheitis (TRT)” [18,19]. This highly contagious disease had limited treatment options apart from the use of antibiotics to limit secondary bacterial infections. Several bacterial and viral pathogens have been linked to this disease syndrome in turkeys including *Alcaligenes faecalis* [20,21], *Bordetella avium* [22], *Escherichia coli* [23], adenoviruses [24], coronavirus [23,25], and infectious bursal disease virus [26]. However, the primary etiological agent was confirmed to be of viral origin in South Africa in 1978 and was initially termed “Turkey Rhinotracheitis Virus” (TRTV) [27]. Virus particles were found to be pleomorphic in shape measuring from 40 nm to 500 nm, including spherical and filamentous forms, with relatively long, regularly spaced fine surface projections [19,28]. The nucleocapsid of the unclassified TRTV was approximately 14 nm in diameter with a helical pitch of 6.5 nm, resembling the analogous structure observed for RSV rather than other paramyxoviruses [28,29]. Analysis of the structural proteins produced in TRTV infected cells showed a close relationship to that of RSV and pneumovirus of mice (PVM), strengthening the hypothesis that this newly discovered virus was the first pneumovirus identified in an avian species [28,30,31].

The novel pneumovirus first identified in South African turkey farms, was soon also identified in farms throughout Western Europe, Asia, and South America [32,33,34,35]. Confirmation of Koch’s postulates following virus isolation and in vivo infection studies provided conclusive proof that this novel virus was the causative agent of TRT [36,37]. It was quickly revealed that chickens (*Gallus domesticus*) were also susceptible to this newly identified virus [38,39]. Although respiratory symptoms were in general milder, the observation of swollen infraorbital sinuses, a distinctive pathognomonic sign of TRT in chickens, resulted in the more common use of the term “swollen head syndrome” for this disease syndrome, though this feature is not pathognomonic to the disease in chickens. A substantial drop in egg production is also apparent in both turkeys and chickens with TRT, thus posing a substantial threat in the poultry industry [40]. The name “Turkey Rhinotracheitis Virus” quickly became outdated with the recognition that this disease also occurs in chickens and that the avian rhinotracheitis observed in virus-infected turkeys is clinically indistinguishable from other respiratory syndromes. This has collectively resulted in the more common use of the name “avian pneumovirus” (APV) [41,42].

Early studies performed with polyclonal sera reported widespread cross-neutralization and thus did not reveal any antigenic differences between APV strains [43,44]. It was not until the 1990s, upon the introduction of new molecular and serological technologies, that strain differences were identified: Monoclonal antibody testing enabled two distinct APV subtypes to be distinguished in 1993 with the original South African 1978 strain found to be more closely related to a strain isolated in the UK in 1985 than to other strains which had been isolated in the UK and France [36,45]. A second independent group reported similar results with a different set of monoclonal antibodies and APV strains, grouping strains isolated in Italy, Hungary, and Spain together and distinguishing them from other UK strains [41]. Furthermore, in 1994 Juhasz and colleagues compared nucleotide and predicted amino acid sequences of the attachment gene (G) of five different APV strains which also indicated at least two distinct subgroups. British and French isolates formed the first subgroup A, whereas Spanish, Hungarian, and Italian strains formed the second subgroup B [46]. These two subgroups were thus proposed to be named subtypes A and B, according to standard RSV nomenclature [46].

A novel subtype AMPV-C was isolated in the state of Colorado in the United States in 1996 [47,48]. This virus was found on a commercial turkey farm and although it appeared to be quickly eradicated in Colorado [47], it soon spread to many other states including Minnesota, North Dakota, South Dakota, Iowa, and Wisconsin [49,50]. This subtype was later detected in other parts of the world with virus isolates or RT-PCR positive samples obtained from a Muscovy duck (*Cairina moschata*) in France in 1999 [51], in pheasants from a live bird market in Korea in 2005 [52], and in Muscovy ducks and chickens from China [53,54]. A phylogenetic study of full genome sequences has shown that the Eurasian AMPV-C lineages form their own clade compared to the AMPV-C lineage present in the USA, although it is not known if this is due to geographic distance or host tropism [55]. Another subtype, AMPV-D, was isolated in French turkey flocks in 1985 and was antigenically and molecularly divergent to other avian pneumovirus strains. AMPV subtype D has not been detected since 1985 and may therefore be speculated to represent a spill-over of a distinct subtype present in an unidentified wild bird reservoir host species [55,56,57].

**Figure 1:**
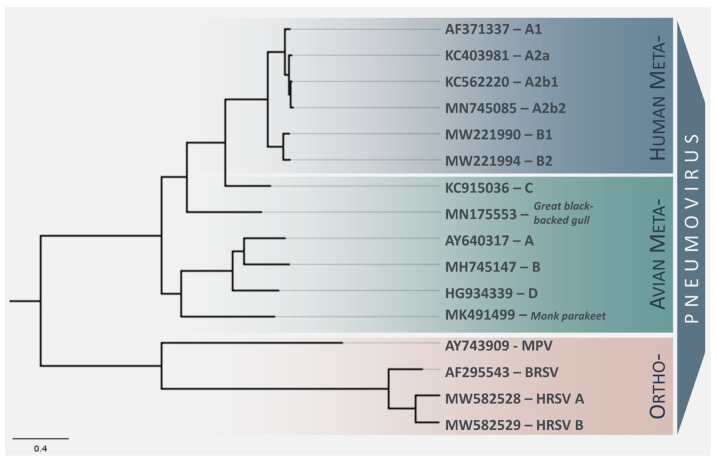
Maximum likelihood phylogeny of metapneumovirus sequences. The phylogenetic tree was constructed using complete genome sequences from NCBI GenBank representing the different subgroups of AMPV and HMPV with orthopneumovirus sequences used as an outgroup. Maximum likelihood phylogeny was performed using a GTR + G model of nucleotide substitution. The tree was generated using bootstrap support of 1000 replications. The tip labels represent the NCBI GenBank accession numbers of respective virus strains, and the scale bar is proportional to the number of nucleotide substitutions per site.

An increased focus in recent years on performing virus surveillance studies via metagenomics resulted in the discovery of two new subtypes of AMPV, forming distinct clades in the phylogenetic tree of metapneumoviruses (Figure 1). An AMPV strain discovered in a monk parakeet (*Myiopsitta monachus*) in 2019 differs from all other recognized subtypes to such an extent that it will most likely be assigned to its own subtype [58]. A second unclassified AMPV subtype was also discovered in 2019 in a great black-backed gull (*Larus marinus*) and is intermediate between AMPV-C and other AMPVs [59]. These two novel strains have not yet been classified by the International Committee on Taxonomy of Viruses (ICTV), but will presumably be named AMPV-E and -F.

## 3. Discovery of Human Metapneumoviruses

It took 23 years after the discovery of AMPV for the second member of the Metapneumovirus genus, HMPV, to be discovered. In 2001, a Dutch group performed virus isolations from clinical samples from 28 epidemiologically unrelated children that had been collected over a time span of 20 years [14]. These children, spanning 0–5 years of age, all shared common characteristics of suffering from a respiratory tract infection (RTI) suggestive of RSV infection for which the most common causes of RTI were ruled out by PCR testing. The distinctive growth phenotype of HMPV in cell culture provided the initial data suggesting that a novel respiratory virus had been isolated. Virus growth in tertiary monkey kidney (tMK) cells was very slow and was only observed in Vero and A549 cells upon the addition of exogenous trypsin to the cell culture medium [14]. The observed cytopathic effects (CPE), which appeared 10–14 days post-inoculation, were analogous to those of RSV infection, displaying large syncytia formation with sudden internal disruption of the multinucleated cells leading to detachment. A virus isolate could cause respiratory tract infection in juvenile cynomolgus macaques and could be recovered from infected animals, thus fulfilling the Koch postulates [14]. Using a molecular technique called random arbitrarily primer PCR (RAP-PCR), unique genetic sequences were identified, and phylogenetic analysis revealed that the new pathogen was most closely related to avian pneumovirus [14]. The same study revealed that the discovered HMPV has been circulating in humans for a much longer time period prior to the first isolation in 2001. Retrospective analysis of serological data suggested that this virus has circulated for more than half a century in humans prior to its discovery and that virtually all children are infected by the age of five [14,60,61]. Upon the completion of the full genome sequence of this novel metapneumovirus, it was clear that it was genetically related to avian pneumoviruses, especially to that of subtype C, sharing an amino acid (aa) identity of approximately 80%. However, the two HMPV genes which encoded the small hydrophobic (SH) and attachment glycoproteins (G) showed little amino sequence identity with those of AMPV subtypes [14].

Following the discovery of HMPV in the Netherlands in 2001, other research groups worldwide also reported the presence of this virus in clinical samples, including in North America, Australia, and Europe [62,63,64,65,66]. Genetic subtyping performed via phylogenetic analysis of the nucleocapsid (N) and attachment glycoprotein (G) gene sequences showed that the virus strains formed two distinct clades with each comprised of two subgroups [67,68,69]. The ICTV later designated the genetic lineages of HMPV, as A1, A2, B1, and B2 which separated into two antigenic subtypes A and B [70]. It was estimated that the most recent common ancestor of HMPV-A and HMPV-B lineages to date back around the years 1964 and 1970 [15]. More recent studies have further divided the subtypes into five genotypes: A1, A2a, A2b, B1, and B2, mainly based on nucleotide variation in the G gene, the most variable gene with respect to sequence identity between HMPV strains [68,71,72]. Novel variants carrying a 111 nucleotide (nt) or 180 nt duplication in the G gene (G-dup) have recently emerged, forming a new clade known as A2b1 and A2b2 (or A2c) [73,74]. However, a recent phylogenetic analysis of whole genome HMPV sequences demonstrated a separate nomenclature indicating that A2b2 and A2c are the same subtype, implying the need for a novel evaluation of nomenclature by the ICTV [75]. The G-dup variants (A2b) are deemed to become the most predominant in hospitalized patients worldwide, first detected in Spain, then Japan, Croatia, and China, suggesting that these novel variants may become the predominant variants worldwide [73,74,76,77,78,79]. No differences in clinical symptoms were observed, although it is to be presumed that variant A2b strains display increased rates of virus transmission [80]. The subtype A1 has not been detected in the most recent molecular surveillance studies, suggesting that this subtype has been outcompeted by strains belonging to other subtypes, particularly strains belonging to the rapidly evolving A2b and A2c clades [81,82].

## 4. Metapneumovirus Taxonomy

Metapneumoviruses were originally assigned by the ICTV to the subfamily Pneumovirinae within the family Paramyxoviridae, but this taxonomic assessment was revised in 2016 with AMPV and HMPV reclassified as members of the genus Metapneumovirus in the newly created family Pneumoviridae within the order Mononegavirales [83]. In the context of both the older and more recent taxonomic classification, metapneumoviruses comprise a separate genus to orthopneumoviruses, such as RSV, due to the absence of the non-structural proteins NS1 and NS2 and a different gene order [83]. According to the ICTV, the demarcation criteria for specific metapneumovirus species are distinguished based on the host of the virus rather than evolutionary divergence with metapneumovirus subgroups distinguished by antigenic and sequence relatedness. This is reflected in metapneumovirus phylogeny as AMPV-C and HMPV form their own distinct clade in comparison to the AMPV subtypes-A, -B, and -D, indicating that AMPV-C and HMPV share a common ancestor, presumably of avian origin (Figure 1) [15]. It is important to note that sequence and antigenic relatedness do not consistently distinguish between HMPV and AMPV. Thus far, the ICTV has not defined a species demarcation cutoff based on evolutionary distance. Brown and colleagues have proposed to change the classification of metapneumoviruses into type I (HMPV and AMPV-C) and type II (AMPV-A,-B and -D) metapneumovirus [55]. Although not yet formally classified by the ICTV, the novel Gull Metapneumovirus detected in 2019 shares an ancestor with AMPV-C and HMPV (“Type I”). In contrast to this, a metapneumovirus detected in a Monk parakeet forms a separate clade with AMPV-A,-B, and -D (type II). We expect the taxonomic classification of metapneumoviruses to continue to develop with the discovery of further novel subtypes of AMPV within the genus Metapneumovirus.

## 5. Metapneumovirus Genome Structure and Protein Function

Members of the genus Metapneumovirus have a non-segmented RNA genome of negative polarity and possess eight genes in the order 3′-N-P-M-F-M2-SH-G-L encoding for nine proteins since the M2 gene contains two open reading frames encoding for the M2-1 and M2-2 proteins [14,84]. The nucleoprotein (N) encapsidates viral RNA and, together with the phosphoprotein (P) and the large polymerase protein (L), forms the ribonucleoprotein complex which mediates virus replication and transcription. The matrix protein (M) aids in the assembly and budding of the virus. The M2-1 and M2-2 proteins regulate virus replication and transcription [85,86]. Metapneumoviruses possess three membrane glycoproteins: the attachment glycoprotein (G), the small hydrophobic protein (SH), and the fusion (F) protein. These viral proteins may act as molecular determinants of host specificity, given their critical roles in virus entry and cell-to-cell spread.

### 5.1. The Attachment Glycoprotein (G)

The metapneumovirus G protein is a highly glycosylated mucin-like type II membrane glycoprotein, consisting of the transmembrane domain and the ectodomain. The role of the metapneumovirus G protein is poorly understood, although it is postulated to play a role in viral attachment to host cells and in antagonizing the host’s innate immune response [87,88]. However, in contrast to the attachment protein of paramyxoviruses, expression of the HMPV G protein from recombinant parainfluenza type 1 in the hamster model did not induce protective neutralizing antibodies [89]. Elucidation of the function(s) of the G protein is dependent on the choice of in vitro model system. A recombinant HMPV lacking the G protein (rHMPV-delta G) has identical growth kinetics to the unmodified virus in LLC-MK2 cells, indicating that the G protein is not essential for virus entry and spread in transformed cells [90]. However, such viruses have been shown to be attenuated in both the hamster and African green monkey models [90,91]. An analogous study performed with AMPV-C lacking the G protein (rAMPV/CO-deltaG) also reported no impediment on virus growth in vitro, but this virus was found to be attenuated in the turkey model [92]. Studies investigating the evolutionary dynamics of AMPV and HMPV have demonstrated higher substitution rates for the G gene (3.5 × 10^−3^ nucleotide substitution per site per year) than for the N gene (9 × 10^−4^ nucleotide substitution per site per year) or F gene (7.1 × 10^−4^ to 8.5 × 10^−4^ nucleotide substitution per site per year) [4,93]. The G protein also displays the highest level of inter-strain diversity with a mean of only 63% aa identity between HMPV subgroups [94]. An even higher level of divergence is apparent between the G protein of AMPV subgroups in which a maximum of only 31.2% amino acids are homologous, with the highest level of diversity present in the cytoplasmic ectodomain [57].

The length of the HMPV G protein is 217 to 241 aa long (Table 1) [94], but in recent years new strains have emerged with a 111 nt or 180 nt duplication within the G open reading frame [73,74]. These strains are often referred to as variant members of the A2b subgroup. The effect of these duplicated sequences on the virus phenotype is still poorly understood. However, the rapid global spread of variant A2b strains suggests increased transmission rates, thus enabling an evolutionary advantage for these emerging variants. A recent study by Piñana and colleagues has postulated that HMPV G proteins containing a duplicated sequence might facilitate immune evasion by enhancing the level of protrusion over the F protein, thus acting as a shield against immune surveillance [80]. A similar phenomenon has also been described with HRSV in which duplicated sequences of 60 bp and 72 bp have been noted in the 2nd hypervariable region of the G ORF of lineages BA and ON1, respectively [95,96]. These lineages have now become predominant worldwide, again suggesting that such duplicated sequences in the G ORF confer an inherent transmission advantage.

AMPV strains have also been shown to contain G proteins of varying lengths. The size of the G ORF of AMPV-A, -B, and -D are reported to be 1176 nt (391 aa), 1245 nt (414 aa), and 1170 nt (389 aa), respectively (Table 1) [55]. The length of the G-protein has also been reported to be variable between virus strains belonging to the AMPV-C subgroup [55,97,98,99]. Notably, this phenomenon has been described for AMPV-C strains originating from the USA in which G ORFs have been reported to be 783 nts, 1321 nts, or 1798 nts [97,98,99]. These size differences are caused by deletions located in the ectodomain at the C-terminus of the G protein. For the “Colorado” strain of AMPV-C, the length of the G gene was initially reported to be 783 nts but was later revealed to be 1798 nt [100]. The mechanism(s) underlying this variability in the G ORF and potential consequences for virus fitness are still not completely understood. One hypothesis is that AMPV-C strains originating in wild birds might have lost part of the G gene ectodomain following infection and subsequent sequential passage through turkeys [100]. To investigate these phenomena, studies have been conducted investigating the serial passage of AMPV-C isolates in cell culture. Velayudhan and colleagues have reported that the sequential passage of clinical AMPV-C isolates derived from domestic turkeys in Vero cells results in truncation of the G protein [100]. The presence of mixed virus populations with respect to the length of the G ORF at low and high passage levels suggested that G protein truncations also occurred following virus replication in domestic turkeys [100]. In contrast, fifty passages of a different AMPV-C isolate originating from a wild bird (aMPV/Canada goose 15a/01) on Vero cells did not result in G protein truncations [101]. Analysis of recombinant AMPV-Cs containing a G with 585 aa or a truncated short G of 252 aa in in vitro and in vivo studies has demonstrated that a truncated C-terminal is not essential for virus viability and hypothesize a role for this region in enhancing attachment specificity and host immunity [102].

### 5.2. The Fusion Protein (F)

The metapneumovirus F protein is a trimeric type I membrane glycoprotein of 539 aa (HMPV) or 537–538 aa (AMPV) (Table 1) [84] and mediates fusion of the viral envelope with the host cell membrane and mediates lateral cell-to-cell fusion, leading to syncytium formation in infected transformed cell lines. The F protein is initially synthesized as an inactive precursor F0 that is proteolytically cleaved by host proteases at a monobasic site into the functional disulfide-linked subunits F1 and F2 [103]. Clinical isolates of HMPV require the addition of exogenous trypsin to the cell culture medium to obtain a productive infection in vitro, as the F protein is not cleaved intracellularly [104]. The ability of some laboratory-adapted HMPV strains to grow in the absence of trypsin is due to the acquisition of an amino acid substitution (S101P) in the putative cleavage site motif [105]. The role of trypsin in triggering cell fusion by AMPV F proteins is more uncertain. An AMPV-B strain can be propagated independently of trypsin with cleavage mediated by TMPRSS12 [106]. Cell-to-cell fusion studies have shown that AMPV F proteins either do not require trypsin for induction of cell fusion [107] or that the AMPV-B F protein does require the presence of exogenous cell fusion [108]. Such differences may be due to the passage histories of the AMPV strains from which the F proteins were derived or problems with standardizing the expression of metapneumovirus F proteins in transient plasmid expression systems.

The F proteins of metapneumoviruses are not dependent upon the attachment protein G to mediate fusion of the virus particle with the host cell membrane, meaning that the G protein is thus dispensable for virus growth in transformed cell lines [90,92]. Moreover, the HMPV F protein also has a role in cell surface attachment via binding of a conserved Arg-Gly-Asp (RGD) motif to integrin αvβ1 on the surface of host cells [109]. Similarly, virus binding and cell fusion induced by AMPV-B is modulated by an interaction between a conserved Arg-Asp-Asp (RDD) motif in the F protein and integrin αvβ1 [110]. Analogous RDD and Arg-Ser-Asp (RSD) motifs present in the AMPV-A and aMPV-C F proteins between aa 329–331 also appear to have a role in modulating cell fusion via assumed binding to integrins [110]. In general, the membrane fusion induced by the F proteins of closely related paramyxoviruses occurs at a neutral pH [111]. However, in some studies, fusion at low pH is enhanced in some but not all HMPV strains which contain G294 or K294 in the F protein [112,113]. The requirement of low pH in triggering AMPV F protein-induced cell fusion is also unclear. Wei and colleagues have reported that low pH is not a requirement of AMPV F protein-induced cell fusion [108], whereas a later study showed a required low pH for induced for cell fusion by AMPV-C [107]. The F protein is also hypothesized to be the main virus determinant of host tropism. This is based on a study by de Graaf and colleagues in 2009 in which the growth and tropism of chimeric HMPVs that contained individual genes of AMPV-C were studied in mammalian and avian cell lines. Chimeric HMPV viruses that contained the F protein of AMPV-C replicated more efficiently than HMPV in a quail fibroblast cell line (QT-6) [114].

### 5.3. The Small Hydrophobic Protein (SH)

The SH protein of metapneumoviruses is a type II integral membrane glycoprotein of 174–175 aa for APMV [115] and 177–183 aa for HMPV (Table 1) [72,84] that has been suggested to act as a viroporin, altering membrane permeability [116]. The SH protein appears to be dispensable for replication as a recombinant HMPV lacking the SH (delta SH) gene was only marginally attenuated in in vitro and in vivo models [90,91]. Although a recombinant AMPV-delta-SH can also be rescued, virus titers are reduced in vitro, syncytial plaque size is enhanced, and virus replication in infected turkeys is reduced in comparison to wild-type AMPV [93,117]. The SH protein may also have a role in regulating innate immune responses as infection of human plasmacytoid dendritic cells with a recombinant HMPV-delta-SH induced an increase in the secretion of interferon alpha in comparison to the wild-type virus [118]. It is important to note that every clinical metapneumovirus isolate described thus far has contained an intact SH ORF. However, upon the sequential passage of HMPV in cell culture, the SH gene is prone to develop frameshift or point mutations that ablate the function(s) of the protein and appear to provide a selective advantage in cell culture [119]. The finding that transient expression of the SH protein significantly decreases F protein-promoted fusion activity [116] suggests one mechanism through which the absence of the SH protein may be beneficial to the growth of HMPV in transformed cell lines.

## 6. HMPV Host Tropism

### 6.1. Zooanthroponotic HMPV Infections

HMPV is a human respiratory virus that has no known animal reservoirs, but zooanthroponotic infections have been reported in endangered great apes such as mountain gorillas (*Gorilla beringei beringei*) and chimpanzees (*Pan troglodytes*) [120,121,122,123,124,125]. A full genome sequence of the “Sambana” HMPV strain (GenBank accession number HM197719), a B2 subtype strain, isolated from a female wild mountain gorilla that succumbed during an outbreak of respiratory illness, shows 99.54% sequence identity to that of a Brazilian “STA755” B2 strain (GenBank accession number MG431250) [120,126]. Furthermore, Bayesian phylogenetic analysis shows a close evolutionary relationship between the Sambana-B2-strain and other B2 strains isolated from South Africa [120]. Pathological analyses showed lesions in the upper respiratory tract, suggestive of a viral infection, along with bronchopneumonia linked to secondary infections by *S. pneumoniae* and *K. pneumonia.* The source of infection was not determined, as the HMPV-infected gorilla died early in the outbreak and had not been in contact with park personnel or veterinarians [120]. Further evidence of probable zoo-anthroponotic HMPV infections in non-human primates has come from serological data [127].

### 6.2. Small Mammal Models

In the past two decades of HMPV research, several animal models have been established which show varying degrees of permissiveness to virus replication. The mouse (*Mus musculus*) model has been largely predominant in HMPV studies due to the availability of reagents, easy handling, and lower costs [128]. However, depending on the specific focus of research, it may be considered a model of limited utility due to the absence of a course of disease that approximates natural infection in humans [128]. The mouse model has been useful in elucidating some aspects of viral pathogenesis and in the development of vaccines and antivirals. Two mouse strains that have been shown to be permissive are BALB/c [129,130] and C57BL/6 mice [131,132] which show airway obstruction, hyperresponsiveness, weight loss, ruffled fur, and huddling behavior upon inoculation with HMPV [133,134,135]. However, it must be noted that inconsistency in data does arise, possibly due to variation in the experimental setup. Some studies have reported little to no permissiveness of BALB/c mice [136,137,138]. This may be due to differences in the virus strain and dose used, mouse strain inbreeding, time point of viral detection, and individual handling (reviewed in [128]). The cotton rat (*Sigmodon hispidus*) has also been investigated as a small animal model of HMPV infection, possibly due to its successful use in the RSV field [133,137,139,140]. Several studies have shown cotton rats to be more permissive to HMPV infection than mice and Syrian golden hamsters (*Mesocricetus auratus*) [137,138,139], although infected cotton rats fail to show overt clinical disease [128,133,137,139]. In contrast to the mouse models, cotton rats do show pulmonary histopathology which is similar to that observed in HMPV-infected cynomolgus macaques (reviewed in [128]). Other susceptible small mammals include guinea pigs (*Cavia porcellus*), Syrian golden hamsters, and ferrets (*Mustela furo*) [85,136,137,138,141], which exhibit varying levels of virus replication, clinical presentation, and humoral response, largely dependent on the experimental set-up. Hamsters and ferrets have been shown to support HMPV replication to high titers, resulting in pulmonary histological changes without overt clinical disease and high virus neutralizing antibody levels [136].

### 6.3. Large Animal Models

Cynomolgus macaques (*Macaca fascicularis*), rhesus macaques (*Macaca mulatta*), African green monkeys (AGM, *Chlorocebus aethiops*), and chimpanzees have all been shown to be permissive to HMPV infection [136,142,143]. In 2004, Kuiken and colleagues assessed HMPV infection of six cynomolgus macaques with respect to viral excretion, viral antigen distribution, and associated pathological lesions [142]. Virus replication was restricted to the respiratory tract, with mild, multi-focal erosive and inflammatory changes in conducting airways, and increased numbers of macrophages in alveoli. Virus antigen expression was mostly restricted to the apical surface of ciliated epithelial cells. Clinical findings were subclinical or mild, corresponding to that observed in immunocompetent middle-aged adults [142]. Rhesus macaques display minimal replication of HMPV upon infection, although the permissiveness of cell cultures derived from this species to virus replication suggests that additional host factors restrict in vivo virus infection [136]. In contrast, African green monkeys (AGM) support higher levels of virus replication and display high titers of neutralizing antibodies following infection and have thus served as a model for in vivo studies assessing potential HMPV vaccine candidates [91,136]. Furthermore, antibodies to subgroup A also neutralized HMPV subgroup B, hinting that a serological correlate to the existence of genotypes may not be present [143]. Although pneumonia or bronchiolitis- resembling human illness could not be reproduced in HMPV-infected AGMs, this model represents the best primate model in which to investigate immunity, vaccines, and therapeutics against HMPV infection [128,136]. The first, and only, time chimpanzees were used as an in vivo model of HMPV infection was by Skiadopoulos and colleagues wherein they were found to recapitulate the respiratory disease observed in humans [143]. In this research, 31 animals were studied; of which, 61% were seropositive for HMPV antibodies, indicating that chimpanzees may be naturally infected by HMPV. The seropositive chimpanzees were protected from HPMV reinfection, whereas the seronegative animals developed respiratory symptoms upon infection with HMPV. Furthermore, following virus clearance, chimpanzees were protected against reinfection, independent of the HMPV subtype. For obvious ethical reasons, chimpanzees have not been used as an animal model for HMPV infection since the publication of this study.

### 6.4. Avian Models

Initial experimental infections of turkeys and chickens conducted by van den Hoogen et al. did not result in data to indicate that chickens or turkeys are susceptible to HMPV infection [14]. In contrast, a larger study conducted by Velayudhan and colleagues, in which 2-week-old turkey poults were infected with four HMPV subtypes (then known as A1, A2, B1, or B2), showed that 30–70% of birds had watery to thick mucus discharge and periorbital swelling between four to nine days post-inoculation [144]. Each of the four subtypes caused transient HMPV infection in turkeys, as indicated by clinical presentation, detection of viral RNA in turbinates, and histopathologic examination, but no seroconversion or recovery of infectious HMPV could be demonstrated [114,144]. A third study from 2009 in which turkey poults were infected with an HMPV B1 subtype and AMPV-C, reported replication of AMPV-C but not HMPV [114]. It is not clear why two studies failed to detect HMPV replication in turkey poults, but the passage history of the different strains may have been a contributing factor as well as the age and background of the birds. Future studies could clarify these issues by use of HMPV strains in which the virus sequence is confirmed to be identical to that present in the original patient sample. Next generation sequencing should also be performed on pre- and post-experimental viral sequences from infected birds to clarify if mutations are required for increased rates of virus replication.

## 7. AMPV Host Tropism

Although AMPV is also referred to as the turkey rhinotracheitis virus, all four recognized subtypes have been shown to infect many domestic and wild avian species.

### 7.1. AMPV-A and B

AMPV A and B are the most commonly detected subgroups and mainly infect species of the Order Galliformes, including turkeys and chickens, which can result in major economic losses for poultry farmers. This host tropism has also been reproduced under experimental conditions with differences noted in the clinical signs observed in these species upon AMPV infection [145]. Turkeys display clear respiratory signs, including dry tracheal coughs and mucus secretions, whereas chickens tend to have a milder disease and mainly suffer complications resulting from the effects of secondary bacterial infections [42,146,147]. To date, there are no natural infections of AMPV-A, B reported in ducks [145].

### 7.2. AMPV-C

AMPV-C strains can be divided into two lineages, namely the American turkey strains, known to be the etiological agent responsible for the outbreaks in turkey farms in the USA in the 1990s and early 2000s, and the Eurasian duck strains which mainly infect duck species (Figure 1). A recent experimental study showed that an AMPV-C strain belonging to the American turkey lineage was able to infect turkeys and chickens, while an AMPV-C strain from the Eurasian lineage could infect both turkeys and ducks [145]. In older studies, chickens, Peking ducks (*Anas platyrhynchos domestica*), and Muscovy ducks have displayed seroconversion, but no clinical signs following infection with an American turkey lineage AMPV-C strain [148,149]. The Eurasian lineage strains are known to infect duck species and have caused outbreaks among Muscovy ducks in which respiratory signs and egg drop syndrome were reported in infected birds. An AMPV-C strain that has not yet been classified into one of the two recognized AMPV-C sublineages due to limited genome sequence data, has caused severe respiratory symptoms in chickens in China in 2012 [53]. This remains the only reported outbreak of symptomatic AMPV-C infection in chickens.

### 7.3. AMPV-D

AMPV-D has been isolated twice from turkeys in France in 1985 and has not reemerged since then [57]. Under experimental conditions, AMPV-D has been shown to be well adapted to turkeys, causing clinical signs of infection in inoculated turkeys and contact birds [145]. Inoculated chickens seroconverted, but viral RNA and virus isolation from the trachea was unsuccessful, suggesting limited virus replication in chickens [145].

### 7.4. Role of Wild and Migratory Birds as Reservoir Species for AMPV

Although AMPV was first isolated from turkeys in South Africa in 1978, the true origins of AMPV are unknown. However, the rapid spread of AMPV within the UK and the close relationship of the early UK strains to the original South African strain suggested a role for wild and migratory birds in the transmission of AMPV [150,151]. Following the early outbreaks of AMPV-C in the USA, the role of wild and migratory birds in the transmission of AMPV to domestic poultry was a major focus of investigation. Many wild avian species have been shown to be positive for AMPV RNA including Canada geese (*Branta Canadensis*), house sparrows (*Passer domesticus*), barn swallows (*Hirundo rustica*), and European starlings (*Sturnus vulgaris*) [152]. Studies involving sentinel ducks placed near turkey farms during outbreaks have shown that ducks may harbor infectious viruses [152,153]. Samples obtained from house sparrows and ring-billed gulls (*Larus delawarensis*) in Minnesota and snow geese (*Anser caerulescens*) from Saskatchewan, Canada, were also found to be positive for AMPV-C [50]. Another study in 2002 found positive AMPV swabs in Canada geese and blue-winged teals (*Spatula discors*) [154]. The known geographical range of AMPV in the USA was extended by Turpin and colleagues in 2008 by the detection of AMPV-C RNA in American coots in Georgia and Canada geese in Georgia and Ohio (18568663). This study also detected AMPV antibodies in American coots, American crows, Canada geese, cattle egrets, and rock pigeons in Georgia, South Carolina, Arkansas, and Ohio [155]. In 2011, AMPV-A and AMPV-B were reported to be present in feral pigeons (*Columba livia*) and a plethora of other wild birds in Brazil [156]. AMPV-C was detected for the first time in Europe in 2012 in mallard ducks (*Anas platyrhynchos*), greylag geese, and common gulls via the use of a pan-paramyxo and pneumovirus RT-PCR assay [157] and more recently in Eurasian wigeon (*Mareca Penelope*) (35261311). AMPV-C was also detected in 2017 in Ontario, Canada in wild waterfowl including the American black duck (*Anas rubripes*), American wigeon (*Mareca americana*), blue-winged teal (*Spatula discors*), mallard, northern shoveler (*Spatula clypeata*), wood duck (*Aix sponsa*), and ring-necked duck (*Aythya collaris*) [158]. AMPV-A was detected in 2019 in different wild birds in Brazil, five years after its last detection in commercial flocks in this country [159]. Collectively, these studies show that migratory birds most likely serve as asymptomatic reservoir hosts but may transmit AMPV to domestic poultry.

### 7.5. AMPV Permissiveness in Mammals

Little research has been undertaken into the capacity of AMPV to infect mammals. A single study by Wei and colleagues has shown that AMPV-C can replicate and persist in the lungs of mice for at least 21 days in association with the development of histopathological lesions in lung tissues [160]. These mice showed clinical signs of illness including fever and breathing difficulties from day one to five post-inoculation. The potential of AMPV to infect humans is unknown, but it can be assumed that prior infection with HMPV would afford a degree of cross-protective immunity against such zoonotic infections. A preliminary cross-sectional serosurvey among turkey meat processing workers and control individuals carried out in the USA in 2011 showed that the former had an increased odds of previous infection with AMPV compared to the controls, suggesting that occupational exposure to turkeys is a risk factor for human infection with AMPV [153]. However, it was concluded that more studies are needed to validate these findings, identify possible modes of AMPV transmission, and determine risk factors associated with presumed human AMPV infections.

## 8. Conclusions and Outlook

In recent years, the use of next generation sequencing has become omnipresent in the field of virus discovery and has enabled the characterization of an ever-increasing number of partial and complete genome sequences of novel viruses in wildlife species, domestic animals, and humans. This includes the discovery of a novel swine orthopneumovirus [161] which complements previously described mammalian orthopneumoviruses in the family Pneumoviridae including RSV, bovine respiratory syncytial virus (bRSV), canine pneumonia virus (CnPnV), and Pneumonia virus of mice (PMV) [83]. It is interesting to note that metapneumovirus sequences have only been detected in samples obtained from birds and humans as natural hosts. The absence of any other confirmed mammalian natural host for metapneumoviruses suggests that AMPV may have spread directly from a bird species to humans without infecting an intermediate mammalian host species, ultimately becoming endemic in the human population. Such a scenario is not unlikely given the finding that several RNA viruses, including influenza viruses, are able to directly transmit from an avian to a human host without the necessity for an intermediate mammalian host species. The unique characteristics of the avian immune system [162,163,164,165,166,167] might facilitate the shedding of large amounts of viruses for prolonged time periods and lead to tolerance of many viruses, facilitating virus evolution, dissemination, and cross-species infections [147,157]. Clearly, further research into the dynamics of AMPV infections in wild bird species would be of interest.

Metapneumovirus research continues to encounter considerable technical challenges with respect to virus propagation in cell culture. Considering the data of numerous studies, metapneumoviruses may require several in vitro passages to produce notable CPEs, depending on virus strain or more generally, on the laboratory setup. Passaging of virus isolates in a particular cell line allows the virus to adapt to these artificial surroundings, producing mutant variants that grow more efficiently. Although these mutant laboratory-adapted isolates produce higher titers to use in future experiments, they do not necessarily reflect the phenotype of a circulating wild-type virus. For future metapneumovirus research studies, particular care should be taken that full genome consensus sequences of recombinant viruses are identical to that present in the original clinical swab material from birds or humans. Generally, it is crucial to sequence isolates pre- and post-experiments to define the molecular basis of adaptation to novel hosts. Metagenomics has also facilitated a rapid expansion in the last decade in the number of available metapneumovirus complete genome sequences which notably includes sequences generated de novo from clinical samples such as the novel highly divergent monk parakeet and gull AMPV strains. Collectively, this may necessitate updated molecular clock modeling to reassess the divergence date of AMPV-C and HMPV.

In summary, obtaining a better understanding of the evolutionary origins of HMPV and the barriers to metapneumovirus cross-species infections will require a better understanding of the molecular and phenotypic differences between AMPV-C and HMPV. This will necessitate more rigorous investigations based on the use of virus isolates which retain the consensus sequence present in clinical samples in conjunction with in vitro and ex vivo primary cell models of avian and human respiratory epithelium. Illuminating the mechanisms through which an avian virus evolved to become an endemic human respiratory virus may hold valuable lessons for better understanding how zoonotic viruses are able to circumvent the barriers to cross-species viral infections.

## Figures and Tables

**Table 1:** Nucleotide (nt) and amino acid (aa) length of metapneumovirus glycoproteins.

Virus	Subtype	Strain	Accession Number	Host Species	F		SH		G	
					nt	aa	nt	aa	nt	aa
HMPV	A1	NL/00/1	AF371337	Human	1620	539	552	183	711	236
	A2a	HMPV/AUS/146892777/2003/A	KC403981	Human	1620	539	540	179	660	219
	A2b1	HMPV/USA/C1-718/2005/A	KC562220	Human	1620	539	558	185	687	228
	A2b2	bj0077	MN745085	Human	1620	539	558	185	765	254
	B1	B/NSW/WM2170539/17	MW221990	Human	1620	539	534	177	726	241
	B2	B/NSW/WM0025022/20	MW221994	Human	1623	540	534	177	717	238
	A1	NL/00/1	AF371337	Human	1620	539	552	183	711	236
AMPV	C	GDY	KC915036	Duck ^1^	1614	537	528	175	1758	585
	C	15a	DQ009484	Goose ^2^	1614	537	528	175	1758	585
	C	PL-1	EF199771	Pheasant	1614	537	528	175	795	264
	C	Colorado	AY590688	Turkey	1614	537	528	175	1758	585
	C	Colorado (truncated G)	AY579780	Turkey	1614	537	528	175	759	252
	(F)	GuMPV_B29	MN175553	Gull ^3^	1617	538	687	228	1641	546
	A	LAH A	AY640317	Vaccine	1617	538	528	175	1176	391
	B	LN16	MH745147	Chicken	1617	538	528	175	1245	414
	D	Turkey/1985/Fr85.1	HG934339	Turkey	1617	538	528	175	1170	389
	(E)	PAR-05	MK491499	Parakeet ^4^	1620	539	522	173	1323	439

^1^ Muscovy duck, ^2^ Canada goose, ^3^ Great black-backed gull, ^4^ Monk parakeet.

## Data Availability

Not applicable.
